# Real-time *in vivo* imaging reveals localised Nrf2 stress responses associated with direct and metabolism-dependent drug toxicity

**DOI:** 10.1038/s41598-017-16491-2

**Published:** 2017-11-22

**Authors:** Shiva S. Forootan, Fiona E. Mutter, Anja Kipar, Takao Iwawaki, Ben Francis, Christopher E. Goldring, B. Kevin Park, Ian M. Copple

**Affiliations:** 1 MRC Centre for Drug Safety Science, Department of Molecular and Clinical Pharmacology, Institute of Translational Medicine, University of Liverpool, Liverpool, L69 3GE UK; 2 Institute of Global Health, University of Liverpool, Liverpool, L69 7BE UK; 3 Laboratory for Animal Model Pathology, Institute of Veterinary Pathology, Vetsuisse Faculty, University of Zurich, Zurich, CH-8057 Switzerland; 4 Division of Cell Medicine, Department of Life Science, Medical Research Institute, Kanazawa Medical University, Ishikawa, 920-0293 Japan; 5 Department of Biostatistics, Institute of Translational Medicine, University of Liverpool, Liverpool, L69 3GL UK

## Abstract

The transcription factor Nrf2 coordinates an adaptive response to chemical and oxidative stress characterised by the upregulated expression of cytoprotective target genes. In order to understand the mechanistic relevance of Nrf2 as a marker of drug-induced stress it is important to know if this adaptive response is truly localised in the context of organ-specific drug toxicity. Here, we address this knowledge gap through real-time bioluminescence imaging of transgenic Nrf2-luciferase (Nrf2-luc) reporter mice following administration of the metabolism-dependent hepatotoxin acetaminophen (APAP) or the direct nephrotoxin cisplatin. We detected localised bioluminescence in the liver (APAP) and kidneys (cisplatin) *in vivo* and *ex vivo*, whilst qPCR, Taqman low-density array and immunoblot analysis of these tissues further revealed increases in the expression level of several endogenous Nrf2-regulated genes/proteins, including heme oxygenase 1 (Hmox1). Consistent with the toxic effects of APAP in the liver and cisplatin in the kidney, immunohistochemical analysis revealed the elevated expression of luciferase and Hmox1 in centrilobular hepatocytes and in tubular epithelial cells, respectively. In keeping with the role of reactive metabolite formation in APAP-induced chemical stress, both the hepatotoxicity and localised Nrf2-luc response were ameliorated by the cytochrome P450 inhibitor aminobenzotriazole. Together, these findings show that Nrf2 can reflect highly-localised cellular perturbations associated with relevant toxicological mechanisms.

## Introduction

Drug toxicity is an impediment to the development of urgently-needed new medicines and causes major clinical complications, often resulting in the post-marketing withdrawal of otherwise effective therapeutic agents^1^. Therefore, innovative strategies are required to improve the pre-clinical detection of drug candidates that pose a risk to patients. One emerging approach, inspired by a landmark report from the National Research Council^2^, involves assessing the ability of a compound to trigger one or more stress response pathways that can reflect cellular perturbations linked to a critical endpoint. Such an approach has inspired several projects (e.g. the Tox21 initiative^3^) which aim to screen large libraries of chemical entities in human cell lines equipped with reporters for major stress responses or other relevant biological pathways, with a view to identifying signatures that are reflective of certain toxicity mechanisms. In the context of drug toxicity, relevant stress responses include those triggered by DNA damage, endoplasmic reticulum stress, inflammation and chemical/oxidative stress^4^.

In mammalian cells the major regulator of the adaptive response to chemical/oxidative stress is the transcription factor Nuclear factor erythroid 2-related factor 2 (Nrf2)^5^. Under physiological conditions, Nrf2 binds to Kelch-like ECH-associated protein-1 (Keap1) in the cytoplasm, leading to its ubiquitination and proteasomal degradation. Under conditions of chemical and oxidative stress, however, the interaction between Nrf2 and Keap1 is disrupted, resulting in the accumulation of the former in the nucleus, where it interacts with antioxidant response elements (AREs) and promotes the expression of target genes including Heme oxygenase 1 (Hmox1), Sulfiredoxin 1 (Srxn1) and NAD(P)H dehydrogenase [quinone] 1 (Nqo1). Consistent with this, genetic disruption of the Nrf2 gene lowers the expression of an array of cytoprotective genes^6,7^ and renders mice more sensitive to the adverse effects of many toxic compounds^8^.

Activation of Nrf2 signalling has been demonstrated in animals exposed to many drugs and chemical entities^9^, yet such *in vivo* studies have almost exclusively relied on the analysis of a single tissue relevant to the toxicological insult (e.g. our previous work showing activation of Nrf2 in the livers of mice exposed to the hepatotoxin acetaminophen^10^). However, to fully understand the ability of Nrf2 to reflect the organ-specific perturbations that typically underlie drug toxicities, it is necessary to assess the response of the pathway in non-target tissues. In particular, such knowledge will inform the reliability of extrapolating findings in cell-based reporter assays into a whole body, *in vivo* context. We previously generated transgenic mice (hereafter referred to as Nrf2-luc mice) expressing the OKD48 reporter, which enables real-time monitoring of the Nrf2-driven response to chemical/oxidative stress^11^. The reporter comprises a transcriptionally inactive luciferase-tagged Nrf2 which is under the transcriptional control of endogenous Nrf2 and subject to post-transcriptional regulation by Keap1^11^. We showed that a bioluminescent signal could be detected throughout the body following exposure to the general oxidative stressors sodium arsenite and ultraviolet radiation^11^. Here, in order to assess the ability of Nrf2 to reflect organ-specific perturbations, we have used real-time bioluminescence imaging of Nrf2-luc mice to reveal localised Nrf2-driven stress responses to drug-induced toxicity.

## Results

### Acetaminophen activates hepatic Nrf2 signalling in vivo

**Figure 1:**
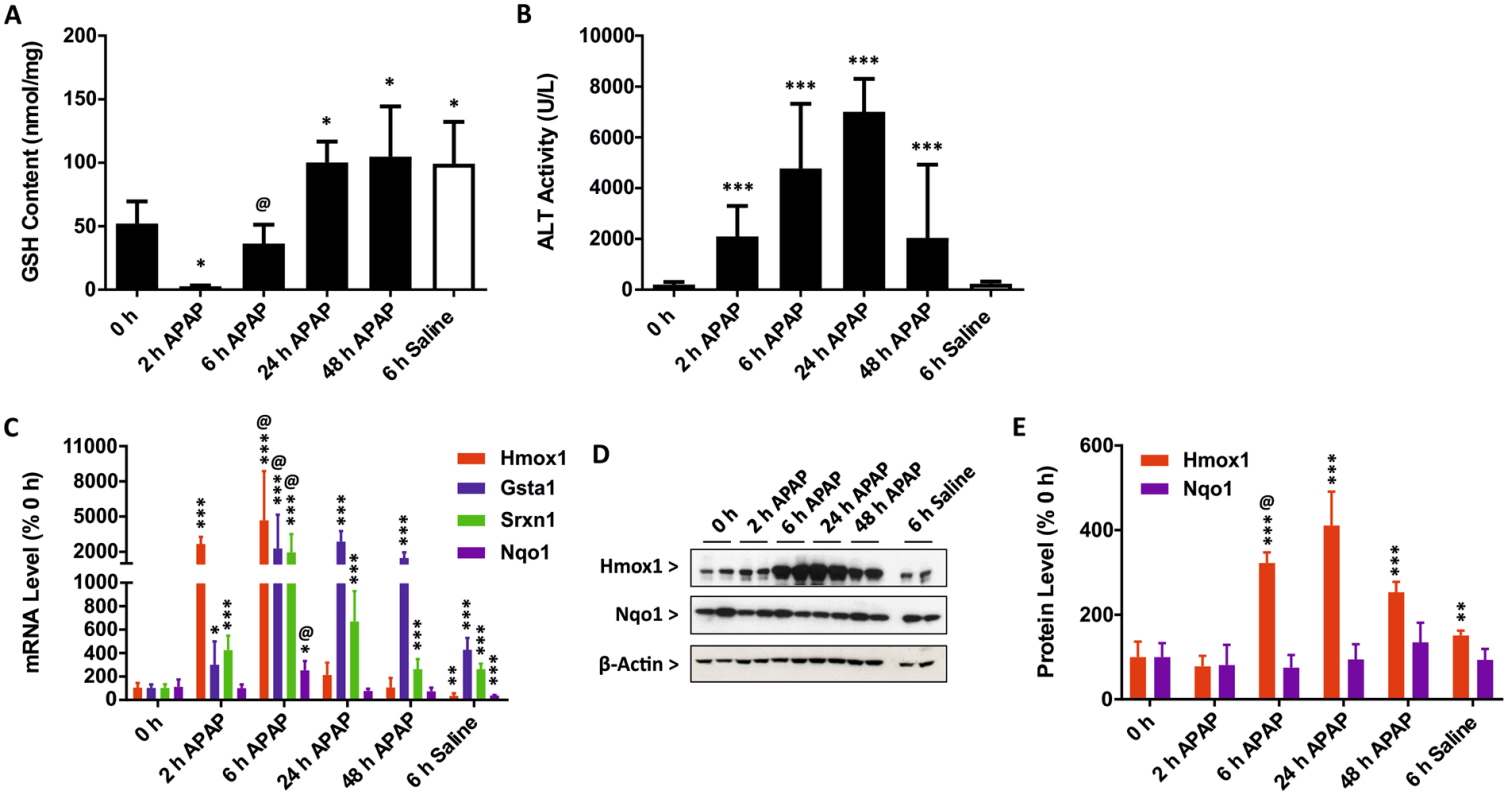
Acetaminophen activates hepatic Nrf2 signalling *in vivo*. Wild type C57Bl/6 J mice (n = 5 per group) were administered saline or 300 mg/kg APAP. (**A**) Total hepatic GSH content and (**B**) serum ALT levels in mice at the indicated times post-APAP administration. (**C**) qPCR analysis of Nrf2 target genes in the livers of mice at the indicated times. (**D**) Immunoblot analysis of Hmox1 and Nqo1 in livers of mice (two representative samples per group) at the indicated times. (**E**) Densitometric analysis of Hmox1 and Nqo1 protein levels in livers of mice (five per group) at the indicated times. Data represent mean + S.D. Statistical analysis was performed with (**A**) one-way ANOVA (Tukey’s multiple comparison) or (**B**,**C**,**E**) a Kruskal-Wallis (Conover-Inman pairwise comparison) test; *P ≤ 0.05, **P ≤ 0.001, ***P ≤ 0.0001 versus 0 h. ^@^P ≤ 0.001 versus 6 h saline.

Our previous work has shown that acetaminophen (APAP) activates Nrf2 signalling in the livers of CD-1 mice^10^. As the transgenic Nrf2-luc mice are of a C57Bl/6 J background, we sought to confirm our earlier findings and establish hepatotoxic conditions in wild type mice of the same strain prior to undertaking bioluminescent imaging studies. Male C57BL/6 J mice were therefore administered 300 mg/kg APAP and culled after 0, 2, 6, 24 or 48 h. Hepatic glutathione (GSH) content was significantly decreased 2 h after APAP administration, followed by a time-dependent rebound (Fig. 1A). Consistent with the rapid and substantial depletion of GSH in the liver, serum alanine aminotransferase (ALT) levels were significantly higher at 2, 6 and 24 h in mice treated with APAP, with recovery evident at 48 h (Fig. 1B). We next assessed the activity of the Nrf2 pathway in the livers of the mice, by quantifying the mRNA expression levels of known target genes. APAP provoked significant increases in the expression of *Hmox1*, *Gsta1, Srxn1* and *Nqo1*, with variable magnitudes and time-dependence of response evident across the four genes (Fig. 1C). The substantial early increases in *Hmox1* mRNA were associated with significant increases in the hepatic protein level of Hmox1 at 6, 24 and 48 h following APAP administration (Fig. 1D,E), whereas the relatively smaller changes in *Nqo1* mRNA were not associated with a significant increase in its protein level (Fig. 1D,E). Together, these data confirm that a hepatotoxic dose of APAP activates Nrf2 signalling in the livers of C57Bl/6 J mice.

### Characterisation of Nrf2-luc mice

In order to ensure that the Nrf2-luc transgene did not alter the basal level of GSH and expression of Nrf2 target genes, we compared these traits in the livers of naïve wild type C57Bl/6 J and Nrf2-luc mice. For both female and male mice, there was no significant difference in hepatic GSH content between the two strains (Fig. S1A). In addition, introduction of the Nrf2-luc transgene did not cause a significant change in the basal expression levels of Nrf2 target genes in the liver (Fig. S1B). The data confirm that the Nrf2-luc transgene does not alter key biochemical pathways *in vivo*.

### Acetaminophen provokes a localised hepatic Nrf2 stress response

**Figure 2:**
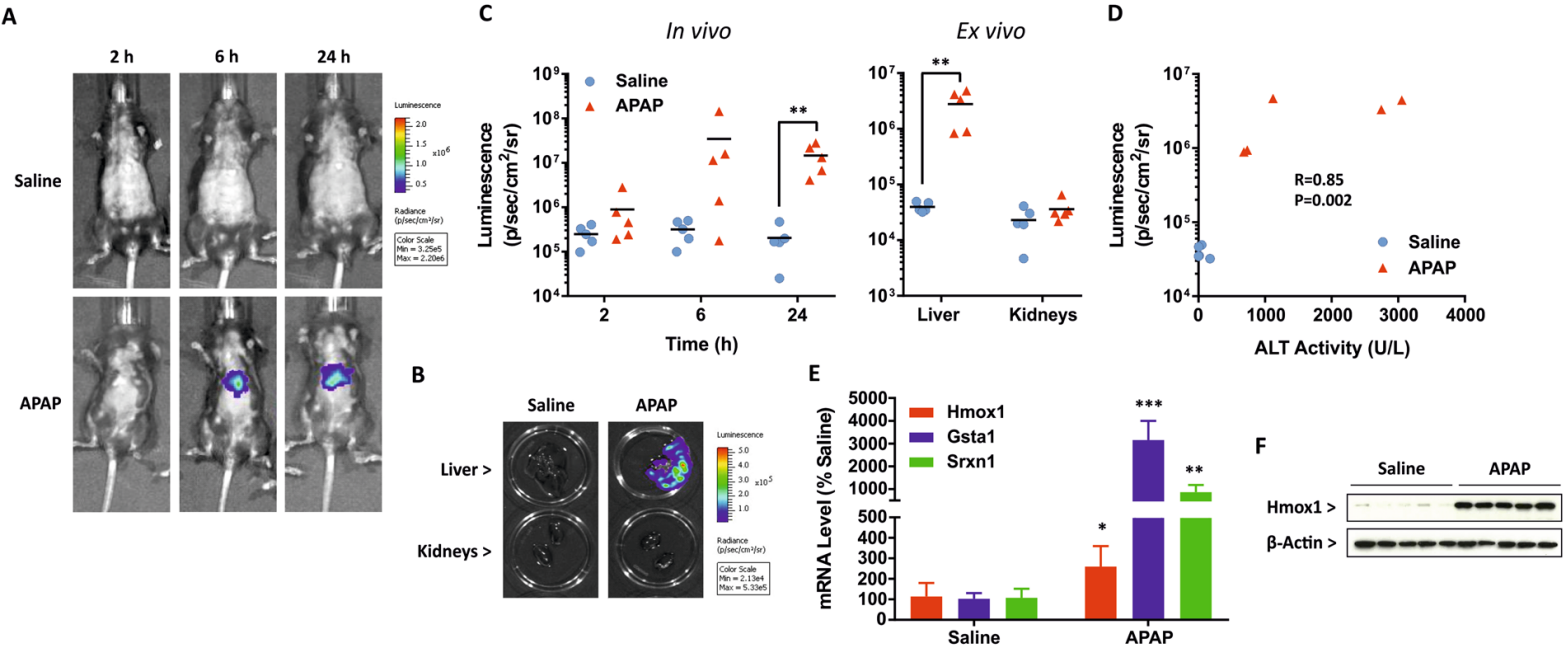
Acetaminophen provokes a localised hepatic Nrf2 stress response. Nrf2-luc mice (n = 5 per group) were administered saline or 300 mg/kg APAP. (**A**) *In vivo* bioluminescence imaging of the same representative mice at the indicated times post-APAP administration. See Fig. S2 for imaging data for all mice. (**B**) *Ex vivo* bioluminescence imaging of livers and kidneys of the mice shown in A, 24 h post-APAP administration. (**C**) Luminescence signals from *in vivo* and *ex vivo* imaging of all mice. (**D**) Correlation of bioluminescence signals in livers imaged *ex vivo* and serum ALT levels in the same mice. (**E**) qPCR analysis of Nrf2 target genes in the livers of mice 24 h post-APAP administration. (**F**) Immunoblot analysis of Hmox1 in livers of mice at 24 h. Data represent mean + S.D. Statistical analysis was performed with a (**C**) Mann-Whitney U test, (**D**) Pearson’s R test or (**E**) unpaired t-test; *P ≤ 0.05, **P ≤ 0.001, ***P ≤ 0.0001 versus saline.

**Figure 3:**
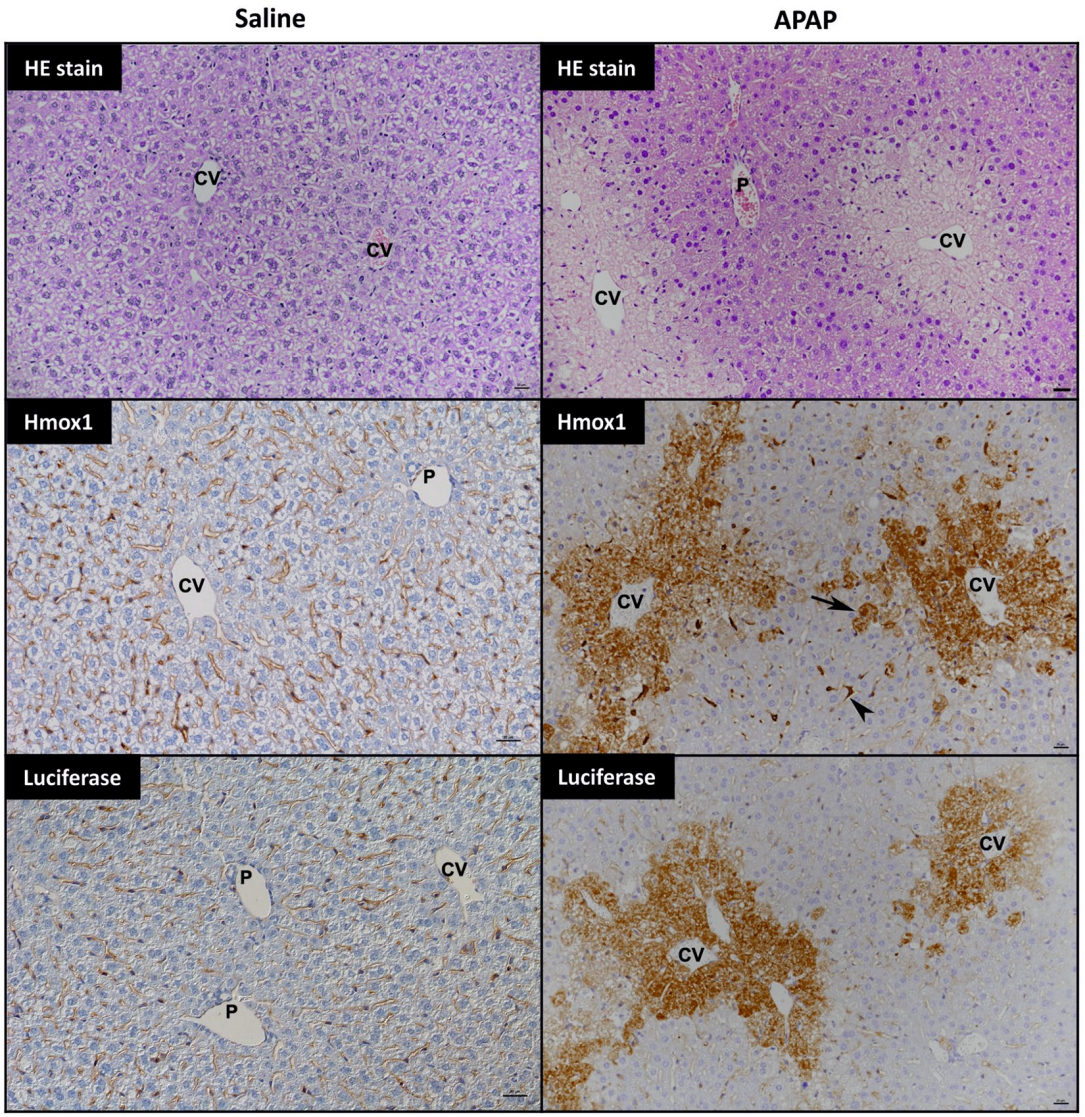
Histological analysis of the hepatic stress response to acetaminophen in Nrf2-luc mice. Nrf2-luc mice were administered saline or 300 mg/kg APAP. At 24 h, in saline-treated mice there were no histological changes (HE stain), Hmox1 expression was restricted to Kupffer cells and erythrocytes within sinuses, and staining for luciferase yielded only a non-specific serum reaction. In APAP-treated mice, there was extensive centrilobular coagulative necrosis with hydropic degeneration of surrounding hepatocytes (HE stain). Hmox1 was expressed by the necrotic centrilobular hepatocytes as well as individual intact hepatocytes adjacent to the affected area (arrow), whilst Kupffer cells close to affected areas also exhibited strong Hmox1 expression. Luciferase was expressed by the necrotic and degenerate centrilobular hepatocytes. CV: central vein; P: portal vein. Scale bars = 20 µm.

Based on our findings in wild type mice, we administered 300 mg/kg APAP or 0.9% saline to female Nrf2-luc mice in order to monitor the Nrf2 stress response in real-time. Whilst no response was evident at 2 h post-dosing (likely due to the time required for transcription and translation of the Nrf2-luc transgene, downstream of the activation of endogenous Nrf2 signalling), *in vivo* imaging detected a localised bioluminescent signal consistent with the anatomical location of the liver in three of five and all APAP-treated mice at 6 and 24 h, respectively (Figs 2A,C and S2). At the latter time point, *ex vivo* imaging confirmed the accumulation of Nrf2-luc in the livers, but not kidneys, of all APAP-treated mice (Figs 2B,C and S2). We did not detect a bioluminescent signal in any of the saline-treated mice (Figs 2A–C and S2). The bioluminescent signals detected in the livers of APAP-treated Nrf2-luc mice *ex vivo* were of variable intensity (Figs 2C and S2), yet there was a significant correlation between the signal intensity and corresponding serum ALT level in the same animal (Fig. 2D), indicating that the intensity of the Nrf2-luc signal can reflect the extent of drug-induced tissue insult. Consistent with the elevated Nrf2-luc reporter activity, Taqman low density array (TLDA) analysis revealed increases in the expression levels of a range of endogenous Nrf2 target genes in the livers of APAP-treated mice 24 h post-dosing (Fig. S3). qPCR analysis confirmed the elevated expression levels of *Hmox1*, *Gsta1* and *Srxn1* under these conditions (Fig. 2E), whilst immunoblotting showed an average 14.5-fold increase (P = 0.008; Mann-Whitney U test) in the hepatic protein level of Hmox1 in Nrf2-luc mice dosed with APAP, compared to vehicle control (Fig. 2F). In keeping with the *in vivo* and *ex vivo* imaging data from Nrf2-luc mice, and in contrast to effects in the liver, there was little perturbation of endogenous Nrf2 target genes in the kidneys of APAP-treated mice 24 h post-dosing (Fig. S4). Finally, and in agreement with the serum ALT measurements, histopathological analysis of the livers of Nrf2-luc mice at 24 h post-dosing confirmed typical APAP-associated hepatic changes, i.e. coagulative necrosis and hydropic degeneration of centrilobular hepatocytes (Fig. 3), with a mean liver injury score of 1.25 (range 0–2.5). Notably, necrotic centrilobular and degenerating hepatocytes exhibited elevated expression of luciferase and Hmox1 in mice treated with APAP, but not saline (Fig. 3). Taken together, these data show that the Nrf2-luc reporter can reflect a localised hepatic stress response to APAP *in vivo* and *ex vivo*.

### Cisplatin provokes a localised renal Nrf2 stress response

**Figure 4:**
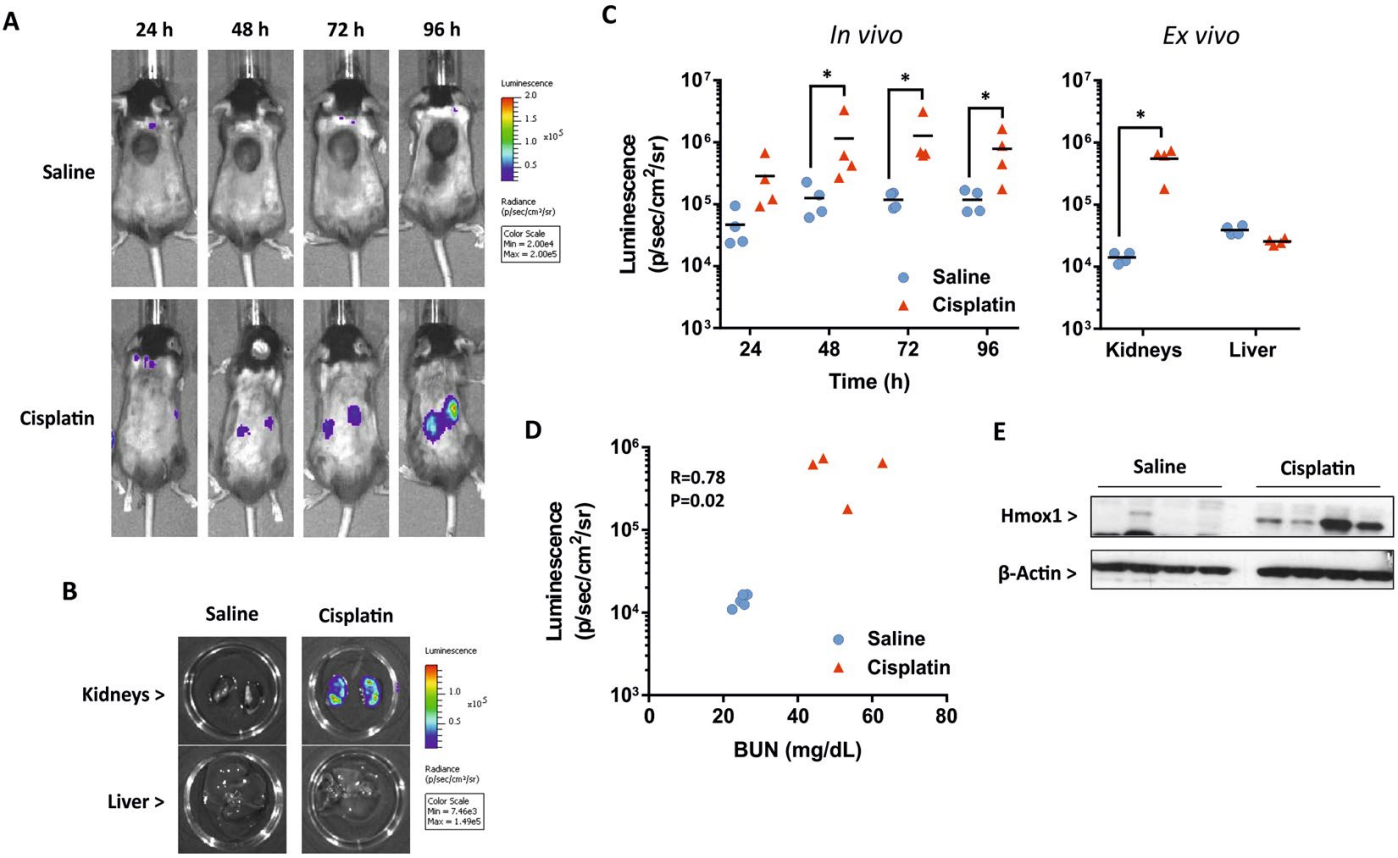
Cisplatin provokes a localised renal Nrf2 stress response. Nrf2-luc mice (n = 4 per group) were administered saline or 20 mg/kg cisplatin. (**A**) *In vivo* bioluminescence imaging of the same representative mice at the indicated times post-cisplatin administration. See Fig. S5 for imaging data for all mice. (**B**) *Ex vivo* bioluminescence imaging of kidneys and livers of the mice shown in A, 96 h post-cisplatin administration. (**C**) Luminescence signals from *in vivo* and *ex vivo* imaging of all mice. (**D**) Correlation of bioluminescence signals in kidneys imaged *ex vivo* and serum BUN levels in the same mice. (**E**) Immunoblot analysis of Hmox1 in kidneys of mice at 96 h. Data represent mean + S.D. Statistical analysis was performed with a (**C**) Mann-Whitney U test or (**D**) Pearson’s R test; *P ≤ 0.05 versus saline.

**Figure 5:**
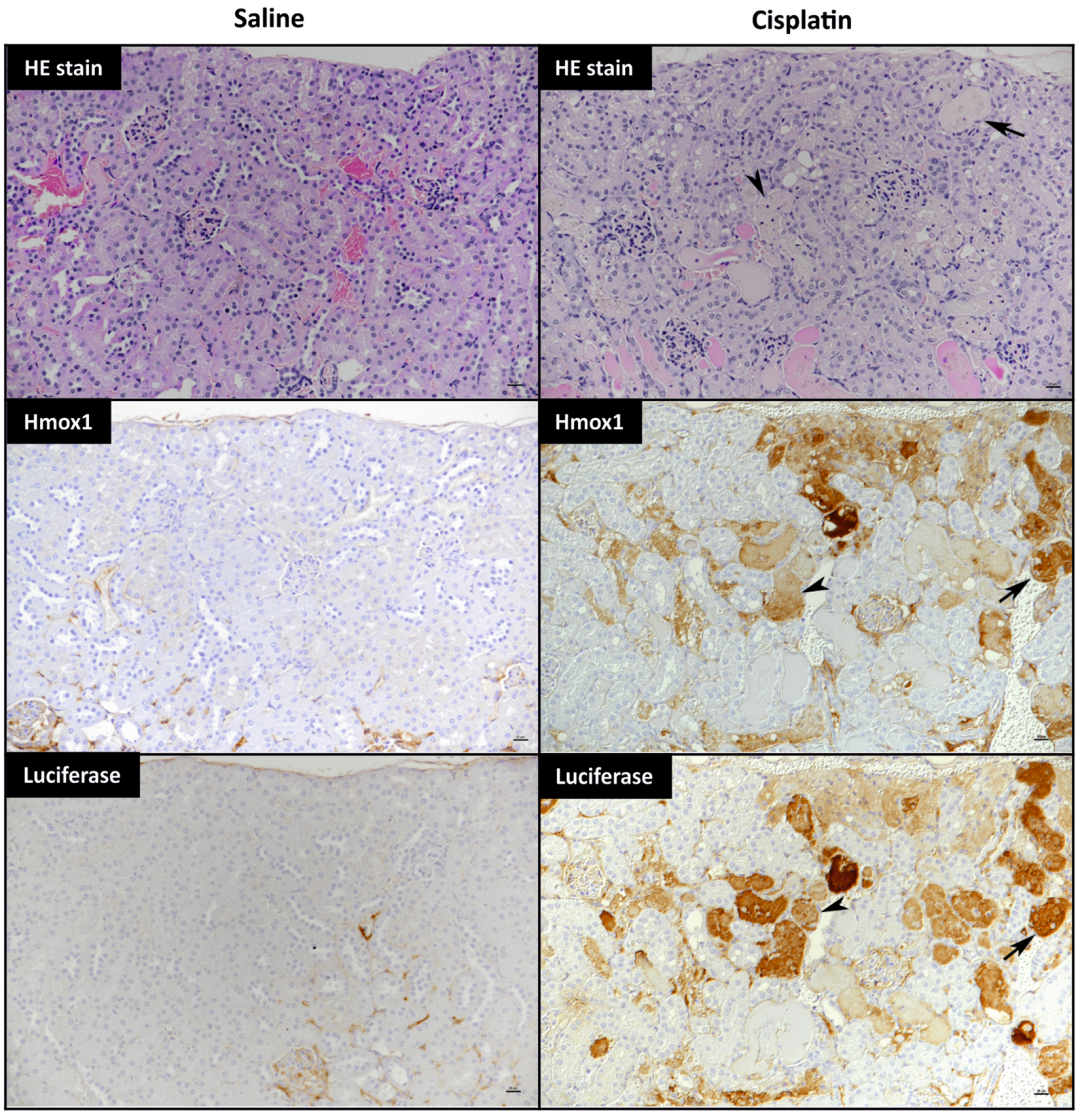
Histological analysis of the renal stress response to cisplatin in Nrf2-luc mice. Nrf2-luc mice were administered saline or 20 mg/kg cisplatin. At 96 h, in saline-treated mice there were no histological changes (HE stain), Hmox1 expression was restricted to intravascular erythrocytes, and staining for luciferase yielded only a non-specific serum reaction. In cisplatin-treated mice, proximal tubules exhibited attenuated epithelium (arrow) or necrosis and loss of epithelial cells (arrowhead), whilst lumina were often filled with protein casts (HE stain). Hmox1 and luciferase expression were detected in viable, degenerating proximal tubular epithelial cells (arrow) and within the proteinaceous material in the lumen of proximal tubules with necrotic epithelial cells (arrowhead). Scale bars = 20 µm.

In order to assess the ability of the Nrf2-luc mice to report on drug-induced stress responses targeting other organs, we treated female Nrf2-luc mice with 20 mg/kg cisplatin to provoke acute kidney injury^12^. In line with the established time course of cisplatin nephrotoxicity, bioluminescence imaging was performed at 24, 48, 72 and 96 h after drug administration and revealed a progressive increase in bioluminescent signal consistent with the anatomical locations of the kidneys (Figs 4A,C and S5). This response was absent in saline-treated mice (Figs 4A,C and S5). *Ex vivo* imaging of the kidneys at 96 h post-dosing confirmed the occurrence of a kidney-specific stress response in all cisplatin-treated animals, with no bioluminescent signal detected in the livers (Figs 4B,C and S5). There was a significant correlation between the intensity of the bioluminescent signals detected in *ex vivo* imaging of the kidneys and blood urea nitrogen (BUN) levels (Fig. 4D), further indicating that the intensity of the Nrf2-luc signal can reflect the extent of drug-induced tissue insult. Whilst relatively small changes in the renal expression levels of endogenous Nrf2 target genes were detected 96 h post-cisplatin administration (Fig. S6), immunoblotting showed an average 8.6-fold increase (P = 0.046; unpaired t test) in the protein level of Hmox1 in the kidneys of cisplatin-treated mice (Fig. 4E). In agreement with the BUN measurements, histopathological analysis of the kidneys of Nrf2-luc mice at 96 h post-dosing confirmed typical cisplatin-induced renal changes, i.e. a variable degree of necrosis and attenuation or total loss of epithelial cells in individual to large groups of proximal tubules (Fig. 5), with a mean kidney injury score of 1.4 (range 0–3). Both luciferase and Hmox1 were expressed by epithelial cells and within the proteinaceous material in the lumen of proximal tubules that contained necrotic epithelial cells in mice treated with cisplatin, but not saline (Fig. 5). Taken together, these data show that the Nrf2-luc reporter can reflect a localised renal stress response to cisplatin *in vivo* and *ex vivo*.

### Generation of albino Nrf2-luc mice

A drawback to using standard C57Bl/6 J mice as the background for studying bioluminescent reporters such as Nrf2-luc is the need to shave the black fur in order to circumvent its ability to suppress the bioluminescent signal. Moreover, standard C57Bl/6 J mice frequently exhibit localised dark skin pigmentation that can interfere with *in vivo* bioluminescence imaging. The impact of the latter trait was clearly demonstrated in the cisplatin study, in which several of the Nrf2-luc mice had a large area of pigmentation covering most of the dorsal skin surface (Fig. S5). In some cases the pigmentation precluded *in vivo* detection of the bioluminescent signal in the kidneys, despite a localised renal Nrf2-luc response being evident from *ex vivo* imaging (Fig. S5). To overcome these limitations and enhance the technical utility of the Nrf2-luc mice, we crossed the original line with B6(Cg)-*Tyr* ^*c-2J*^/J (B6-albino) mice carrying a mutation in the tyrosinase gene, which results in the complete absence of pigment from hair and skin^13^. Following administration of the pharmacological Nrf2 activator sulforaphane, *in vivo* imaging demonstrated the ability to detect a bioluminescent signal without shaving in albino, but not standard, Nrf2-luc mice (Fig. S7). In the latter, a bioluminescent signal was detected only after shaving (Fig. S7). Therefore, subsequent experiments were conducted with albino Nrf2-luc mice.

### The Nrf2 stress response to acetaminophen requires reactive metabolite formation

**Figure 6:**
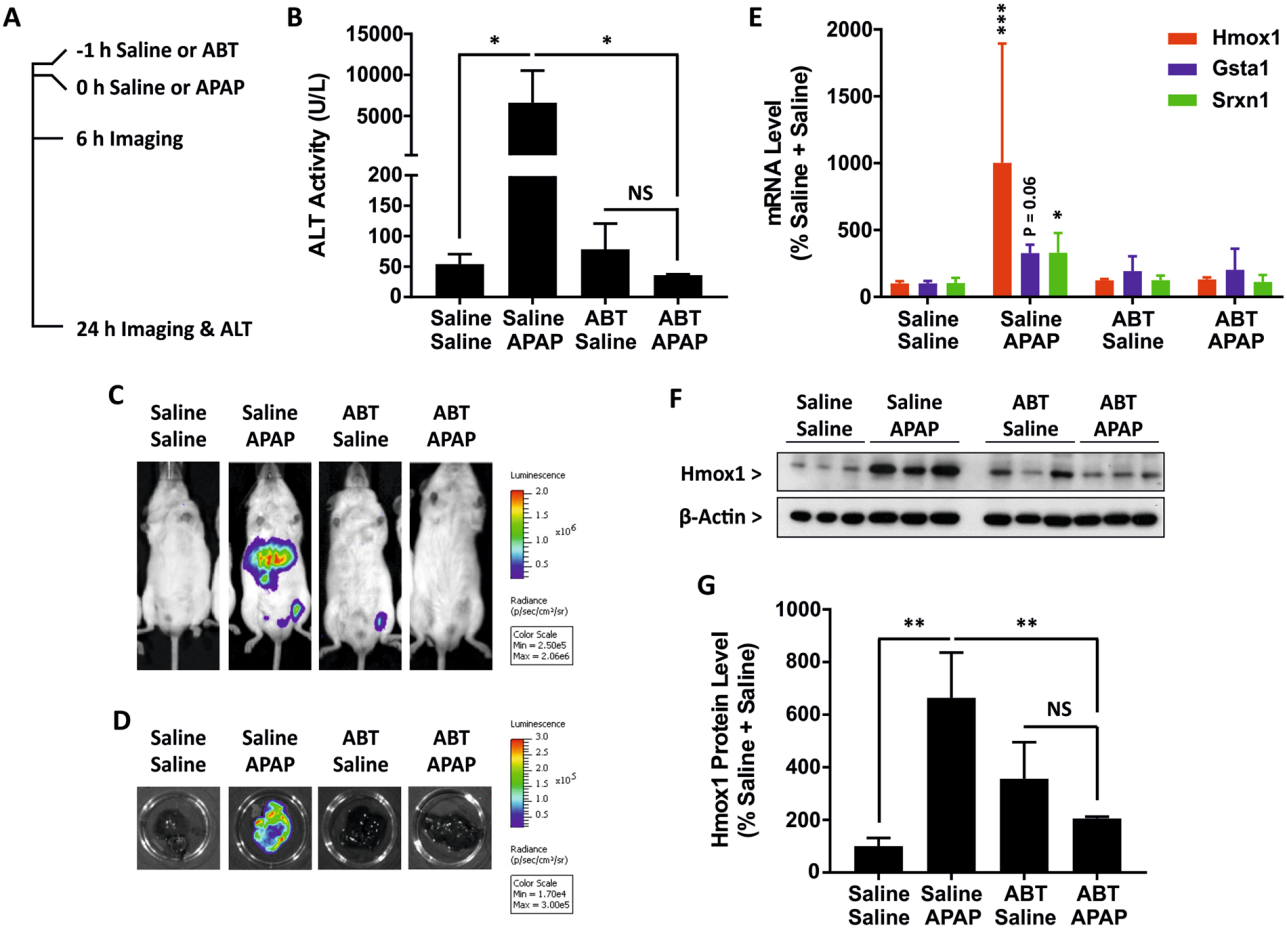
The Nrf2 stress response to acetaminophen requires reactive metabolite formation. Nrf2-luc mice (n = 3 per group) were administered saline or 100 mg/kg ABT, then 1 h later administered saline or 300 mg/kg APAP. (**A**) Overview of study design, with times of drug administration, imaging and serum ALT measurements indicated. (**B**) Serum ALT levels in mice treated as indicated, 24 h post-APAP administration. (**C**) *In vivo* and (**D**) *ex vivo* bioluminescence imaging of the same representative mice, 24 h post-APAP administration. See Fig. S8 for imaging data for all mice. (**E**) Luminescence signals from *in vivo* and *ex vivo* imaging of all mice. (**F**) qPCR analysis of Nrf2 target genes in the livers of mice treated as indicated, 24 h post-APAP administration. (**G**) Immunoblot analysis of Hmox1 protein levels in livers of mice at 24 h. (**H**) Densitometric analysis of Hmox1 proteins levels in G. Data represent mean + S.D. Statistical analysis was performed with (**B**,**H**) one-way ANOVA (Tukey’s multiple comparison) or (**E**,**F**) a Kruskal-Wallis (Conover-Inman pairwise comparison) test; *P ≤ 0.05, **P ≤ 0.001, ***P ≤ 0.001 versus saline + saline or as indicated; NS, non-significant.

**Figure 7:**
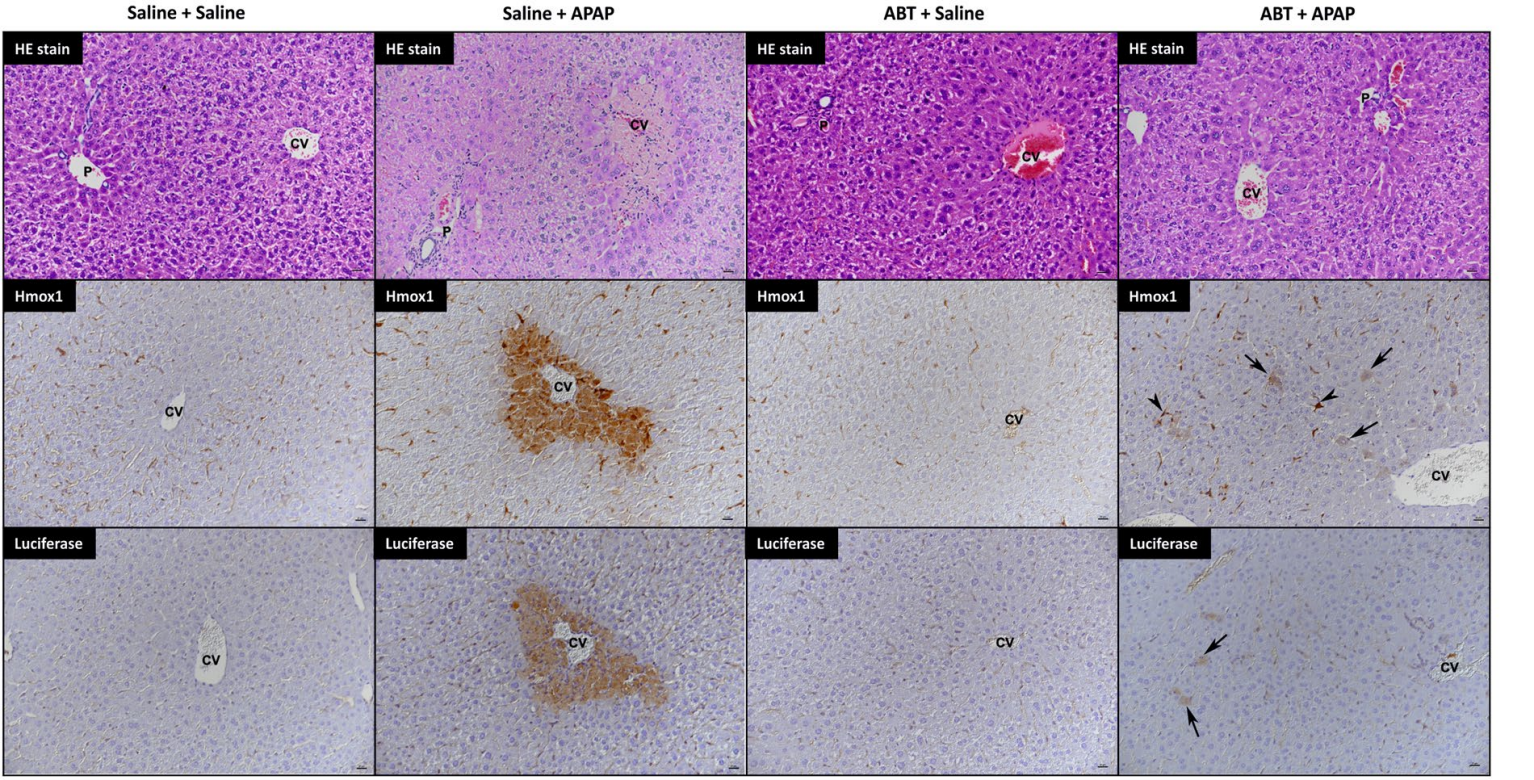
Histological analysis of the effect of aminobenzotriazole on the hepatic stress response to acetaminophen in Nrf2-luc mice. Nrf2-luc mice were administered saline or 100 mg/kg ABT, then 1 h later administered saline or 300 mg/kg APAP. At 24 h, in mice treated with saline + saline, there were no histological changes (HE stain), Hmox1 expression was restricted to Kupffer cells and erythrocytes within sinuses, and staining for luciferase yielded only a non-specific serum reaction. In mice treated with saline + APAP, there was extensive centrilobular coagulative necrosis with glycogen loss (confirmed by PAS reaction, data not shown) of surrounding hepatocytes (HE stain). Hmox1 was expressed by the necrotic centrilobular hepatocytes as well as proximate Kupffer cells. Luciferase was expressed by the necrotic and degenerate centrilobular hepatocytes. The livers of mice treated with ABT + saline showed features identical to those observed in mice treated with saline + saline (see above). In mice treated with ABT + APAP, histological changes (HE stain) were restricted to a slight condensation of centrilobular hepatocytes (equivalent of reduced glycogen content; PAS reaction not shown). Hmox1 expression was seen in random individual and occasional smaller aggregates of morphologically unaltered hepatocytes (arrows). Kupffer cells close to positive hepatocytes also showed enhanced expression of Hmox1 (small arrows). Luciferase expression was detected in random individual and occasional smaller aggregates of morphologically unaltered hepatocytes (arrows). CV: central vein; P: portal vein. Scale bars = 20 µm.

The mechanism underlying APAP hepatotoxicity in preclinical species and humans is known to involve the cytochrome P450 (CYP450) -mediated bioactivation of the parent compound to the reactive metabolite N-acetyl-p-benzoquinone imine (NAPQI), which can covalently modify cellular proteins and provoke mitochondrial dysfunction^14^. To ensure that the localised Nrf2-luc response to APAP reflects this toxicological mechanism, we pre-treated male albino Nrf2-luc mice with 100 mg/kg of the CYP450 inhibitor aminobenzotriazole (ABT) for 1 hour, followed by administration of 300 mg/kg APAP (Fig. 6A). As expected, ABT inhibited the development of APAP hepatotoxicity, with serum ALT levels found to be significantly lower in mice treated with ABT for 1 hour followed by APAP for 24 h, compared to those treated with APAP only (Fig. 6B). In addition, ABT almost completely abolished the histopathological changes induced by APAP in the liver (Fig. 7). Indeed, the only evidence of toxic changes were a few random necrotic hepatocytes (data not shown). Whilst *in vivo* (6 and 24 h) and *ex vivo* (24 h) imaging confirmed that APAP provoked a hepatic bioluminescent response, consistent with findings in the standard Nrf2-luc mice (Fig. 2A,B), pre-treatment with ABT abolished the APAP-induced bioluminescent signal in the livers of albino Nrf2-luc mice (Figs 6C–E and S8). In further support of this, qPCR analysis showed that ABT suppressed the ability of APAP to provoke increases in the hepatic expression levels of Nrf2 target genes (Fig. 6F), whilst the induction of Hmox1 protein by APAP was also found to be sensitive to ABT pre-treatment (Fig. 6G,H). Immunohistochemical analysis confirmed these results; in ABT pre-treated mice, Hmox1 and luciferase expression was only observed in random individual, morphologically unaltered hepatocytes, in contrast to the extensive expression detected in centrilobular hepatocytes in mice treated with APAP only (Fig. 7). Taken together, these data confirm that the Nrf2-driven response to APAP reflects the metabolism-dependent, localised insult that is known to underlie its hepatotoxicity.

## Discussion

The Nrf2-driven response to chemical and oxidative stress has been associated with several forms of drug toxicity in pre-clinical studies^9^. Amongst ongoing efforts to reduce attrition and improve the benefit:risk balance of new drugs, there is an increasing interest in using the perturbation of Nrf2 and other stress response pathways as mechanism-based markers of toxicity^2^. For example, amongst the Tox21 panel of cell lines is a β-lactamase reporter for the Nrf2 response (HepG2 ARE-*bla*)^3^, whilst fluorescent reporter cell lines encompassing several elements of the Nrf2 pathway have been developed through the Innovative Medicines Initiative-supported MIP-DILI consortium^15^. These *in vitro* platforms are particularly suited to high-throughput screening (HTS) of large compound libraries in the early stages of drug discovery, in which the potential of a compound to provoke an Nrf2-driven stress response can be determined, yet they cannot consider the influence of drug distribution on the likelihood of a stress response occurring in a given organ *in vivo*. Therefore, novel *in vivo* platforms that complement *in vitro* HTS assays and allow selected compounds to be investigated in a more holistic manner could enhance our understanding of the mechanisms and risks associated with certain toxicological traits.

In this study, consistent with the established targets of the toxicities in rodents and man, we have shown that the Nrf2 response to APAP and cisplatin is specific to the liver and kidney, respectively. Notably, cisplatin was classified as active in the Tox21 HepG2 ARE-*bla* assay (Fig. S9A), similar to compounds such as diethyl maleate (Fig. S9B) that we have shown to stimulate hepatic Nrf2 signalling following administration to mice^10^. However, we did not detect a bioluminescent response in the livers of cisplatin-treated Nrf2-luc mice, emphasizing the importance of placing *in vitro* findings in a whole-body context in order to confirm the occurrence and organ-specificity of the perturbation, and understand its relevance to *in vivo* toxicological mechanisms.

The capacity to reflect Nrf2-driven stress responses to chemically reactive metabolites represents another advantage of Nrf2-luc mice over existing *in vitro* reporter platforms. In man, APAP liver injury is dependent on the generation and accumulation of the electrophilic quinoneimine NAPQI^14^. We have previously shown that direct application of NAPQI to mouse Hepa-1c1c7 hepatoma cells triggers an Nrf2-driven stress response^16^, yet the relative inability of these and other immortalised liver cell lines to perform certain drug bioactivation reactions^17^ has rendered them unsuitable for investigating the role of reactive metabolite formation in the Nrf2 response to APAP *in vitro*. Here, in keeping with the zonation of the liver lobule and predisposition of centrilobular hepatocytes to generate NAPQI, we have shown that the Nrf2 stress response triggered by APAP occurs predominantly in hepatocytes around the central vein, and that pharmacological inhibition of CYP450-mediated reactive metabolite formation impedes the ability of APAP to provoke both Nrf2 activation and hepatocellular necrosis. These observations show that the Nrf2-driven stress response can reflect highly-localised, metabolism-dependent cellular perturbations associated with relevant toxicological mechanisms.

Alternative reporter mouse models for monitoring the activity of the Nrf2 pathway have been reported previously. For example, Henderson *et al*. generated transgenic mice expressing β-galactosidase under the transcriptional control of the mouse *Hmox1* promoter, and used them to investigate the ability of non-genotoxic carcinogens to provoke oxidative stress in the liver, through post-mortem X-gal tissue staining^18^. Yates *et al*. generated mice expressing luciferase under the transcriptional control of a triplicate ARE sequence cloned from the mouse *Nqo1* gene, enabling the response to triterpenoid Nrf2 activators to be visualised *in vivo* using bioluminescence imaging^19^. Taking an alternative approach, Shuhendler *et al*. used an injectable nanosensor to detect reactive oxygen and nitrogen species in wild type mice, through chemiluminescence and fluorescence resonance energy transfer^20^. Consistent with our findings, the nanosensor was used to demonstrate a reduction in APAP-induced oxidative stress in the livers of mice pre-dosed with ABT^20^.

In keeping with 3Rs principles, the use of real-time bioluminescent imaging to monitor Nrf2-driven stress responses allows each animal to act as its own control and enables longitudinal measurements to be taken, requiring fewer animals per study. Given the limited resolution of bioluminescence imaging, it will not always be possible to definitively assign an *in vivo* signal to an organ/tissue without the use of post-mortem *ex vivo* imaging. However, *in vivo* imaging can guide the decision on when, and with which tissues, to perform *ex vivo* analyses, and can avoid the termination of an experiment at an arbitrary time point when an Nrf2 response is clearly absent. As the proof-of-concept investigations described here have been performed with single toxic doses of APAP and cisplatin, in future studies it will be important to incorporate a range of non-toxic and toxic doses of these and other relevant compounds in order to determine the sensitivity of the Nrf2-luc reporter to subtle forms of chemical and oxidative stress that are not associated with overt organ injury. Rather than be employed as a front-line screening tool, we envisage that Nrf2 reporter mice and other emerging technologies for measuring oxidative perturbations *in vivo* could be used in later stages of toxicity assessment to determine the organ specificity of chemical/oxidative stress responses detected *in vitro* and investigate underlying toxicological mechanisms. These and other innovative approaches can contribute to the improved risk assessment of new drugs.

## Methods

### Materials

Unless stated otherwise, all reagents were from Sigma-Aldrich.

### Animal experiments

All animal experiments were conducted according to the UK Animals (Scientific Procedures) Act 1986 and approved by the University of Liverpool Animal Welfare Committee. Wild type C57BL/6 J mice (6–8 weeks old) were purchased from Charles River Laboratories. Nrf2-luc reporter mice^11^ were bred from pairs of male heterozygote and female wild type mice, and housed in a 12 h dark/light cycle in a temperature and humidity controlled, specific pathogen-free environment. Mice were fed CRM (P) diet (Special Diets Services) ad-libitum. To generate albino reporter mice, Nrf2-luc mice were backcrossed onto the B6(Cg)-*Tyrc-2J*/J (B6-albino) strain (Jackson Laboratory) for two consecutive generations of breeding. Genotyping of ear snips was performed by Transnetyx Inc. using a real-time PCR assay and primers specific to Firefly luciferase. For APAP studies, following overnight (16 h) fasting, mice were administered 300 mg/kg APAP or 0.9% saline (vehicle control) via intraperitoneal (IP) injection. Alternatively, mice were administered 100 mg/kg ABT or saline via IP injection 1 h prior to APAP administration. For cisplatin studies, mice were administrated 20 mg/kg cisplatin or saline via IP injection. Sulforaphane (50 mg/kg) was administered via IP injection. Following bioluminescence imaging (see below) mice were culled via exposure to increasing concentrations of carbon dioxide or via IP injection of 1000 mg/kg Pentoject (Animalcare). For each mouse, half of the right liver lobe and one entire kidney were fixed in 4% paraformaldehyde (PFA; pH 7.4) for histological examination, with the remaining liver tissue and kidney flash frozen. Blood was collected via cardiac puncture and allowed to clot for 30 min at room temperature (RT). Serum was isolated via centrifugation to enable analysis of liver and kidney injury biomarkers.

### Bioluminescence imaging

Nrf2-luc mice were imaged using an *In Vivo* Imaging System (IVIS) (PerkinElmer), under anaesthesia with isoflurane. Mice were injected IP with 150 mg/kg D-luciferin (Promega). After 5 min, mice were placed in the IVIS chamber and data were collected and analysed using Living Image software (Xenogen) according to the manufacturer’s instructions. Quantification of luminescence signals was achieved using the Region of Interest (ROI) function within Living Image software. Within a single experiment, an area was drawn around the broadest signal and used for all other animals in that experiment. Total flux within the ROI was considered as the signal intensity. For *ex vivo* imaging, mice were culled, tissues excised and immersed in 300 µg/mL D-Luciferin and analysed using the IVIS platform. For quantification of *ex vivo* luminescence signals, an area was drawn around the dish containing the tissue.

### Alanine aminotransferase assay

Serum ALT levels were measured using Infinity ALT Liquid Stable Reagent (Thermo Fisher Scientific), according to the manufacturer’s instructions.

### Glutathione assay

Total GSH levels were measured in liver tissues as previously described^21^.

### Blood urea nitrogen assay

Serum BUN levels were measured using a Quantichrom Urea Assay kit (BioAssay Systems), according to the manufacturer’s instructions.

### Histology and immunohistochemistry

After PFA fixation for 24–48 h, liver and kidney specimens were trimmed and routinely embedded in paraffin wax. Consecutive sections (3–5 µm) were prepared and routinely stained with haematoxylin and eosin (HE), underwent the Period Acid Schiff (PAS) reaction, or were subjected to immunohistochemical staining. For immunohistochemistry, an autostainer (Dako) was used. Briefly, sections were dewaxed, dehydrated and subjected to antigen retrieval (20 min incubation at 98 °C in citrate buffer, pH 6 for Hmox1, and in EDTA buffer, pH 9 for luciferase). After incubation with the primary antibodies (mouse anti-Hmox1, MA1-112, Thermo Fisher Scientific; mouse anti-firefly luciferase, ab16466, Abcam) for 1 h at RT and blocking of endogenous peroxidase (peroxidase block; Dako) for 10 min at RT, sections were incubated for 30 min at RT with the detection system (Envision System HPR Mouse; Dako), followed by incubation with diaminobenzidine as chromogen and counterstaining with haematoxylin. Liver and kidney injury were assessed using previously reported histopathological scoring systems^22,23^. All histological and immunohistochemical specimens were examined by a veterinary pathologist (AK) who was blinded to the treatment of the animals.

### Taqman low-density array analysis

TLDA cards containing probes for established Nrf2 target genes were generated by Applied Biosystems. A pool of all samples was used as a calibrator across cards, with individual gene expression levels normalised to the housekeeping gene 18 S ribosomal RNA. Analysis was performed on an ABI ViiA 7 Thermocycler, as previously described^7^.

### cDNA synthesis and qPCR analysis

Total RNA was extracted from 30 mg of tissue using an RNeasy Mini Kit (Qiagen). RNA quantity and purity were determined using a Nanodrop 1000 Spectrometer (Thermo Fisher Scientific). RNA was reverse transcribed to cDNA using GoScript Reverse Transcription System (Promega) according to the manufacturer’s instructions. qPCR analysis was performed using Power SYBR Green (Thermo Fisher Scientific) on an ABI ViiA 7 Thermocycler (Applied Biosystems) according to the manufacturer’s instructions. Primer sequences for mouse *Hmox1*, *Gsta1*, *Srxn1*, *Nqo1* and *Gapdh* are detailed in Supplementary Table 1. For each sample, the average threshold cycle (Ct) value was normalized to *Gapdh* and the relevant control sample, using the formula 2^−∆∆Ct^.

### Western blot analysis

Western blot analysis of Nrf2 targets in liver or kidney tissues was performed as previously described^24^. Uncropped blots are provided in Fig. S10. The Hmox1 (ab13243), Nqo1 (ab2346) and β-actin (ab6276) antibodies were from Abcam. Band intensities were quantified using ImageJ.

### Statistical analysis

Pearson’s R correlations and associated P values were calculated using the R software package hmisc^25,26^. All other statistical analyses were performed using GraphPad Prism 5.0 (GraphPad Software). Differences were considered significant at P < 0.05.

## Electronic supplementary material

Supplementary material
