# Supplementary material for: Real-time in vivo imaging reveals localised Nrf2 stress responses associated with direct and metabolism-dependent drug toxicity
Source: Sci Rep. 2017 Nov 22;7:16084. doi: 10.1038/s41598-017-16491-2 (PMC5700176; doi:10.1038/s41598-017-16491-2)
Supplement: Supplementary file 1 — Supplementary material [file 41598_2017_16491_MOESM1_ESM.pdf]

Supplementary Material

Real-time *in vivo* imaging reveals localised Nrf2 stress responses associated with direct and metabolism-dependent drug toxicity

Shiva S. Forootan, Fiona E. Mutter, Anja Kipar, Takao Iwawaki, Ben Francis, Christopher E. Goldring, B. Kevin Park, Ian M. Copple.

Gene	Primer	Sequence
Mouse *Hmox1*	Fwd	5′- GTC AAG CAC AGG GTG ACA GA -3′
	Rev	5′- ATC ACC TGC AGC TCC TCA AA -3′
Mouse *Gsta1*	Fwd	5′- CAG CCT GGC AGC CAG AGA -3′
	Rev	5′- TCT GTG GCT CCA TCA ATG CA -3′
Mouse *Nqo1*	Fwd	5′- TTT AGG GTC GTC TTG GCA AC -3′
	Rev	5′- GTC TTC TCT GAA TGG GCC AG -3′
Mouse *Srxn1*	Fwd	5′- AAA GTG CAG AGC CTG GTG -3′
	Rev	5′- CTT TGA TCC AGA GGA CGT CG -3′
Mouse *Gapdh*	Fwd	5′- TGT CCG TCG TGG ATC TGA C -3′
	Rev	5′- CCT GCT TCA CCA CCT TCT TG -3′

Table S1 – Primer sequences for qPCR analysis of Nrf2 target genes.

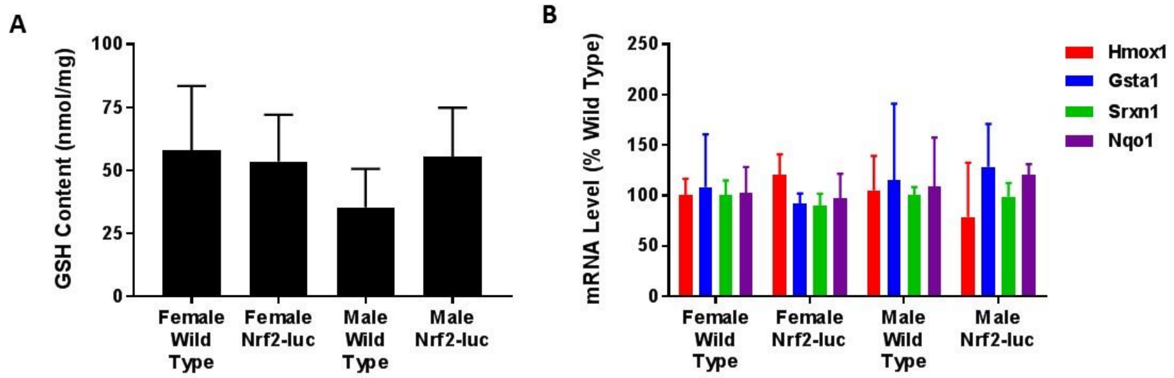

Fig. S1 - Characterisation of Nrf2-luc mice. (A) Basal hepatic GSH content in female and male wild type (C57Bl/6J) and Nrf2-luc mice. (B) qPCR analysis of the basal expression levels of Nrf2 target genes in the livers of female and male wild type and Nrf2-luc mice. Female and male data are expressed as a percentage of female and male wild type, respectively.

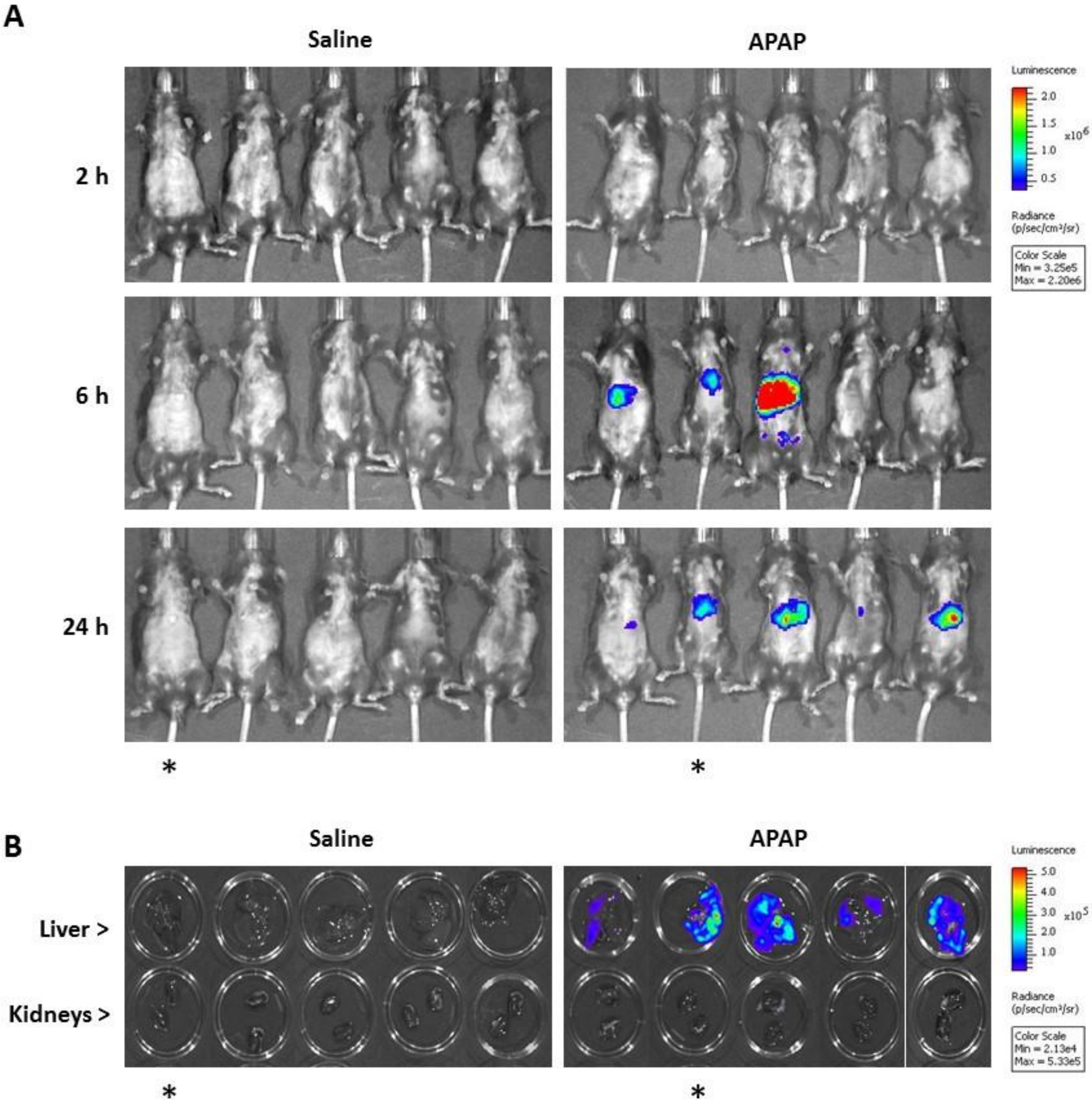

Fig. S2 - *In vivo* & *ex vivo* imaging of all Nrf2-luc mice treated with saline/acetaminophen. Nrf2-luc mice (n=5 per group) were administered saline or 300 mg/kg APAP. (A) *In vivo* bioluminescence imaging of mice at the indicated times post-APAP administration. (B) *Ex vivo* bioluminescence imaging of livers and kidneys of the mice shown in A, 24 h post-APAP administration. * = representative mice/tissues shown in Fig. 2.

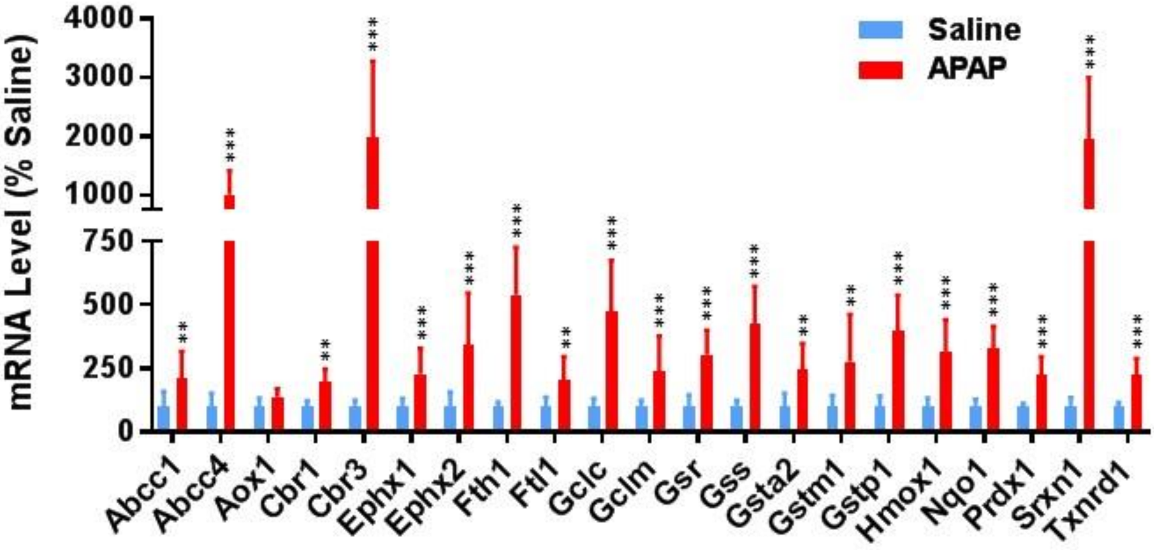

Fig. S3 – Endogenous Nrf2 target gene expression levels in the livers of Nrf2-luc mice treated with saline/acetaminophen. Nrf2-luc mice (n=5 per group) were administered saline or 300 mg/kg APAP. After 24 h, hepatic Nrf2 target gene expression levels were determined using customised Taqman low-density array cards. Genes significantly increased in response to APAP were identified using a Kruskal-Wallis (Conover-Inman pairwise comparison) test; ** $P \leq 0.001$, *** $P \leq 0.0001$ versus saline.

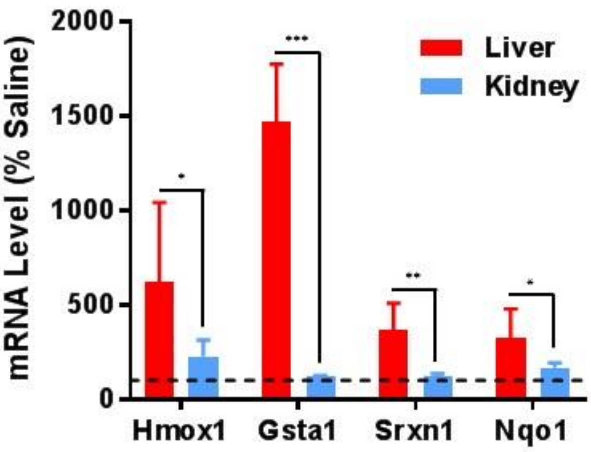

Fig. S4 - Endogenous Nrf2 target gene expression levels in the livers and kidneys of mice treated with acetaminophen. Wild type mice (n=5 per group) were administered saline or 300 mg/kg APAP. After 24 h, Nrf2 target gene expression levels were determined in livers and kidneys by qPCR. Significant differences between gene responses to APAP in liver and kidney were identified using an unpaired t-test; * P≤0.05, ** P≤0.001, *** P≤0.0001.

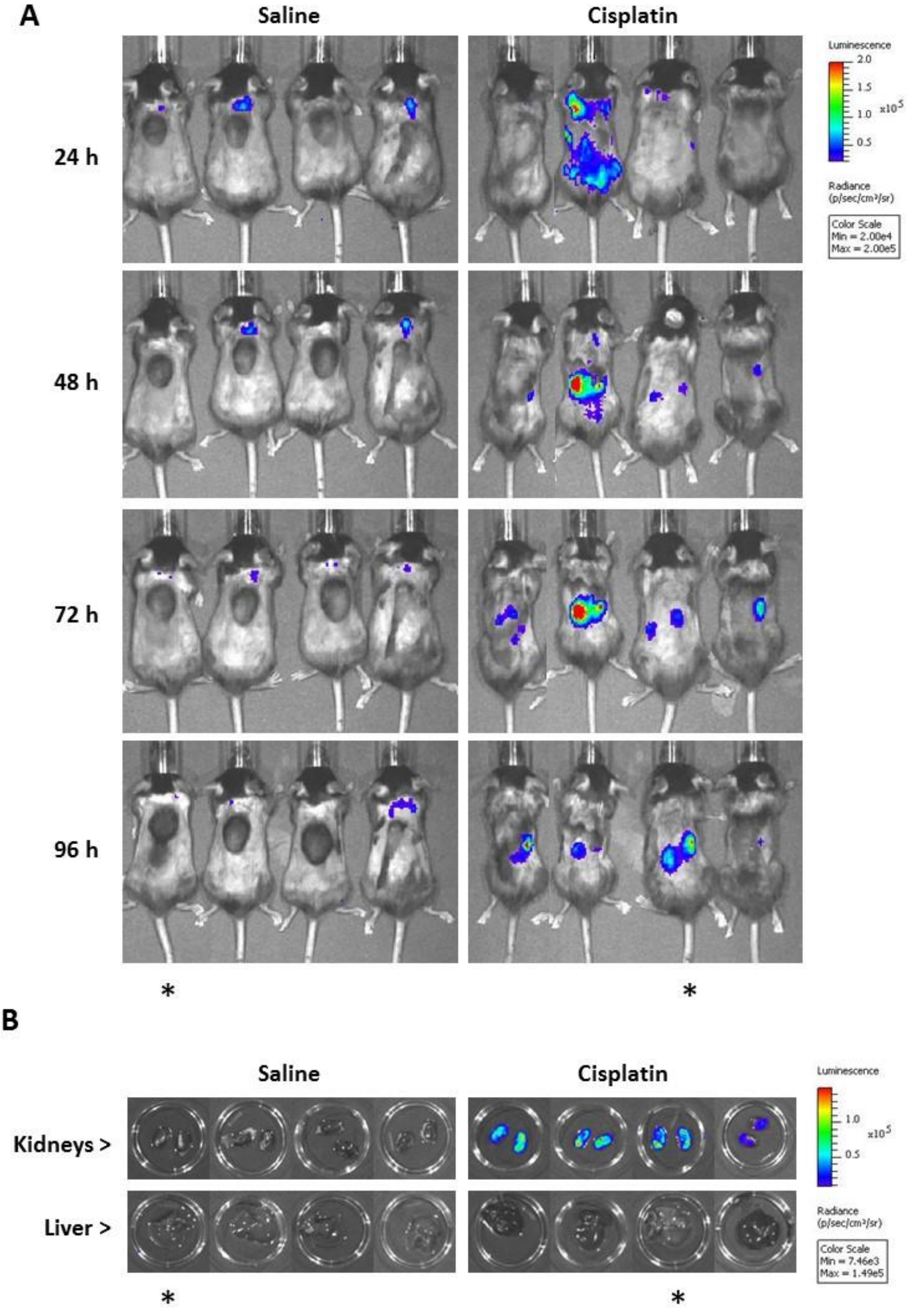

Fig. S5 - *In vivo* & *ex vivo* imaging of all Nrf2-luc mice treated with saline/cisplatin. Nrf2-luc mice (n=4 per group) were administered saline or 20 mg/kg cisplatin. (A) *In vivo* bioluminescence imaging of mice at the indicated times post-cisplatin administration. (B) *Ex vivo* bioluminescence imaging of kidneys and livers of the mice shown in A, 24 h post-cisplatin administration. * = representative mice/tissues shown in Fig. 4.

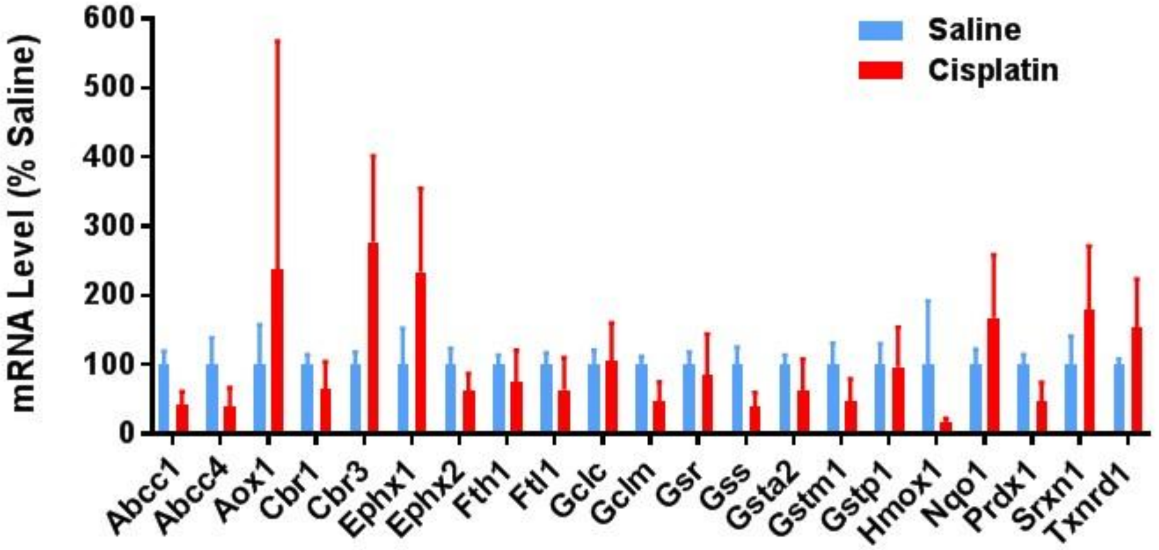

Fig. S6 – Endogenous Nrf2 target gene expression levels in the kidneys of Nrf2-luc mice treated with saline/cisplatin. Nrf2-luc mice (n=4 per group) were administered saline or 20 mg/kg cisplatin. After 96 h, renal Nrf2 target gene expression levels were determined using customised Taqman low-density array cards. No genes were significantly increased in response to cisplatin, according to a Kruskal-Wallis (Conover-Inman pairwise comparison) test.

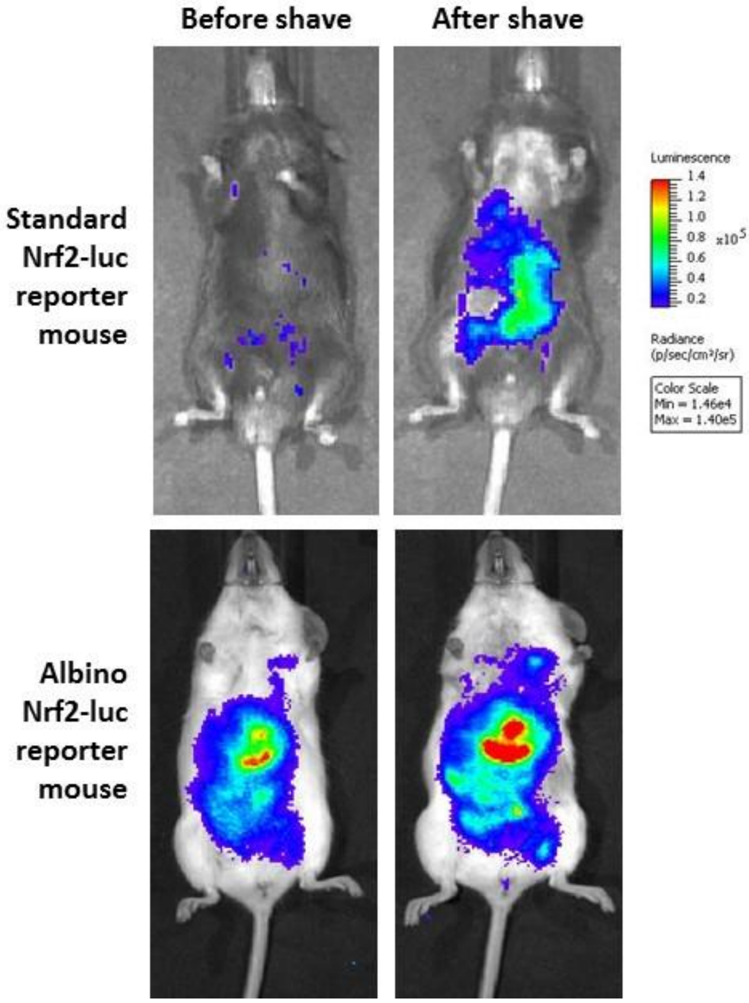

Fig. S7 - Imaging of sulforaphane treated black & albino mice pre/post-shaving. Standard and albino Nrf2-luc mice were administered 50 mg/kg sulforaphane. After 6 h, *in vivo* bioluminescence imaging was performed on unshaved mice. The mice were then shaved and immediately re-imaged. In contrast to the albino Nrf2-luc mouse, a bioluminescent signal was detected in the standard Nrf2-luc mouse only after shaving.

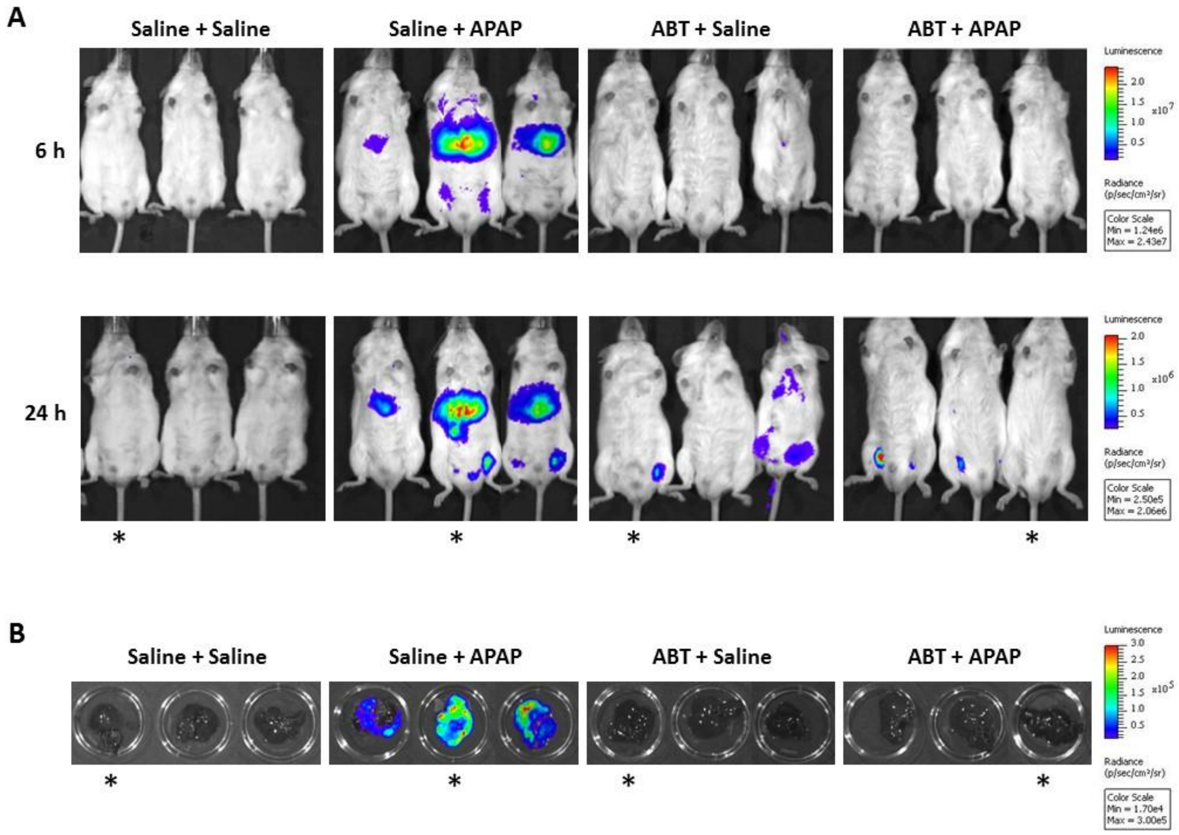

Fig. S8 - *In vivo* & *ex vivo* imaging of all Nrf2-luc mice treated with acetaminophen -/+ aminobenzotriazole. Albino Nrf2-luc mice (n=3 per group) were administered saline or 100 mg/kg ABT, then 1 h later administered saline or 300 mg/kg APAP. (A) *In vivo* bioluminescence imaging of mice at the indicated times post-APAP administration. (B) *Ex vivo* bioluminescence imaging of livers of the mice shown in A, 24 h post-APAP administration. * = representative mice/tissues shown in Fig. 6.

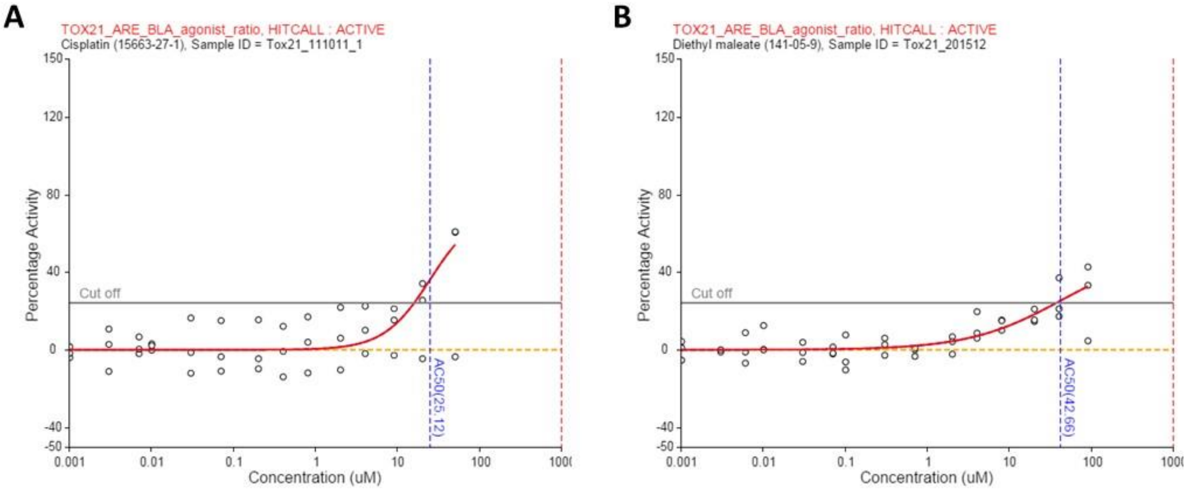

Fig. S9 - Tox21 HepG2 ARE-*bla* assay data. Active responses in HepG2 ARE-*bla* cells exposed to the indicated concentrations of (A) cisplatin or (B) diethyl maleate for 24 h. Data accessed through the ToxCast Dashboard (https://actor.epa.gov/dashboard).

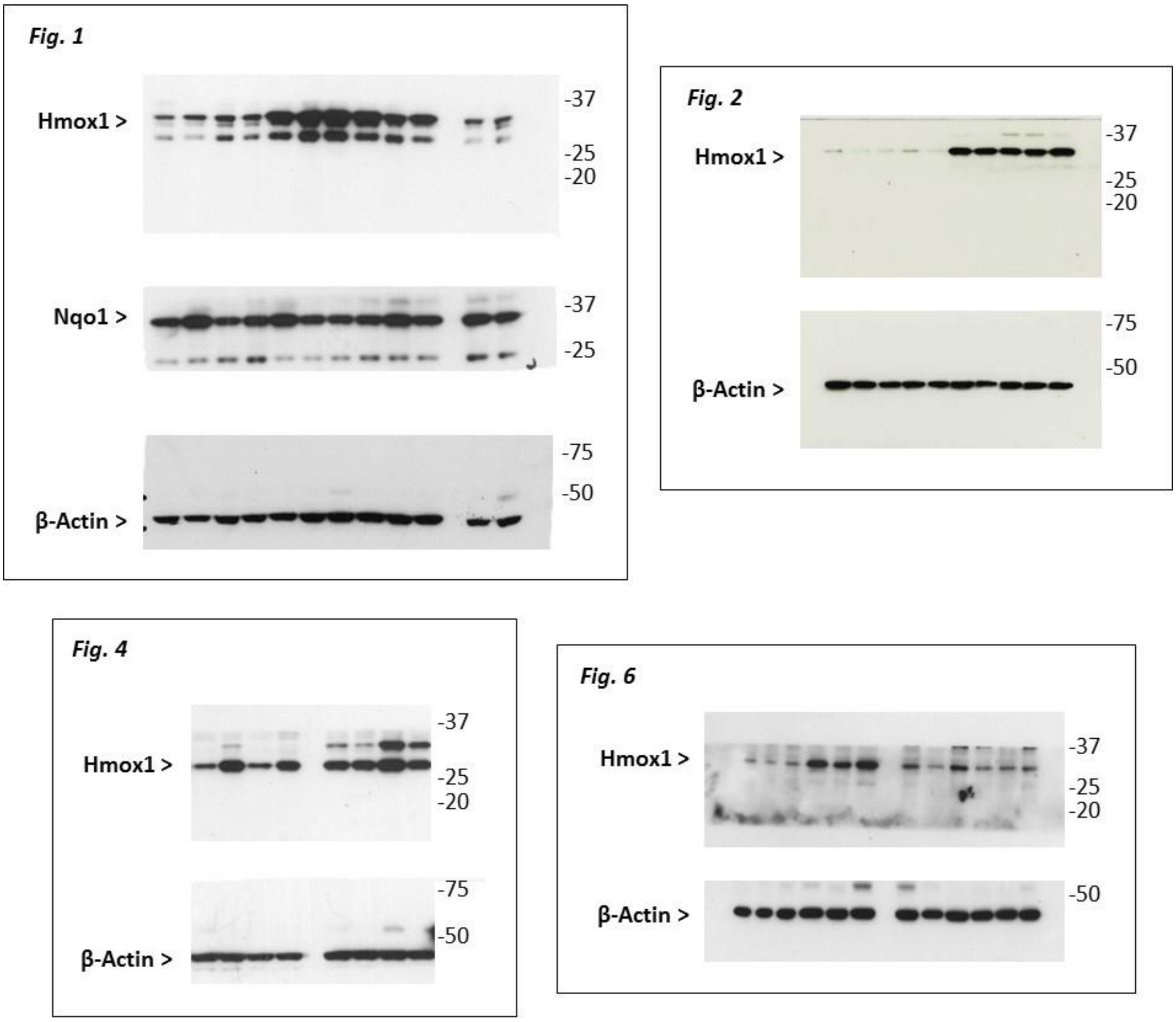

Fig. S10 – Uncropped immunoblots for data shown in the indicated figures. Relevant molecular weights are labelled in kDa.
